# Miscellaneous

**Published:** 1848-07-01

**Authors:** 


### Miscellaneous.

Frequent occurrence of Gangrene of the Lungs among the Insane.—In the course of six years, 1840—1845, there were examined in the Pathological Theatre at Prague, 3437 bodies, of which 3102 were from the hospital, and 335 from the asylum for lunatics. Among the former, gangrene of the lungs occurred fifty-five times—viz., in 1·6 per cent. of the cases; in the latter, twenty-five times—viz., 7·4 per cent. This proportion is the more to be relied on, from its being very nearly the same in each of the six years referred to.—*Präger Vierteljahrschrift,* 1847.

Demonomania.—Demonomania seldom occurs in early life, and it is seldom cured; recovery, however, does sometimes take place, as in an instance of late occurrence — a young woman, aged twenty-one, in whom a fright brought on religious despondency, succeeded by demonomania. She conceived that "five or six devils had entered into her, and caused her to renounce the Lord—that she was possessed by Satan, and was the devil." She would stand for hours together looking at her nails, occasionally objected to take her food, and had a disposition to put an end to herself. By the employment of laxative medicines and the douche (the affusion of cold water upon her head when in the warm bath) she was, at the end of ten months, completely restored to reason. Demonomaniacs are in general emaciated—have an expression of great distress—love solitude—sleep little, and occasionally attempt to commit suicide. They are sometimes very insensible to bodily suffering—they can bear to have pins thrust into them without appearing to feel them. Females are more frequently the subjects of religious insanity than males: it is in general difficult of cure.—*Morison's Physiognomy of Mental Diseases.*

Monomania with Fear.—Fear forms the characteristic feature of a variety of partial insanity of frequent occurrence. Those who labour under it are afraid of one or more objects, or they have a dread of everything, in which case the term *panaphobia* is employed to denote the disorder. In some cases there is a vague and undefined terror; frequently delusions or erroneous ideas of objects and sounds occur. These delusions probably have a relation to ideas with which they had previously been familiar; for instance, the occurrence of fires has given rise to insanity, with excessive dread of being burnt: a lady of fortune used to spend the night in being driven in her carriage through the streets of London, afraid lest her house might take fire. The fear of damnation has often been the leading feature of insanity in persons of a religious turn of mind; fear of poverty occurs in some who have, by industry, accumulated a good fortune. Among the objects of fear in the insane, are poison, robbers, prison, and the police; persons in this complaint are inclined to interpret everything to their own disadvantage, to exaggerate their feelings, and sometimes to ascribe imaginary crimes to themselves. In consequence of the irritation under which they continually labour, they are generally emaciated and feeble; from their fear of doing wrong, they are undecided and restless, and cannot make up their minds to do the most common acts of life—repeated attempts are made before they accomplish those of eating, drinking, relieving nature, going to bed or rising up—occasionally attempts are made to commit suicide, but these generally fail, on account of their fear and indecision. Of the causes which produce this variety, the emotion of fear itself sometimes gives rise to the disorder; it, however, occurs in insanity originating from other sources: females and young persons are most liable to it.—*Ibid.*

Causes of Insanity.—Insanity (says Dr. Male, in his "Epitome of Juridical Medicine") may arise from various causes, as blows or injuries inflicted on the head or brain; excessive indulgence of the passions of lust, anger, and revenge; intemperance; repelled discharges; fanaticism; intense study; mortified pride, and disappointed ambition. Grief and despair, arising from supposed irretrievable misfortunes, frequently occasion this calamity. It is well known, indeed, that the number of insane persons was greatly increased in France by the horrors and misery induced by the Revolution. I believe (says Dr. M.) a frequent cause of madness is suffering the mind to dwell too long on one particular train of thought, whether the subject be real or imaginary. The ideal lucubrations with which many, particularly young persons, allow their minds to be amused, and stray in the regions of fancy, called castle-building, or day-dreaming, I conceive weakens the mind, and, abstracting it from real and useful objects, absorbs its energies in fanciful and futile speculations, which often lead to insanity. Dr. J. Johnstone, in his work on Madness, also says, "It may readily be supposed that a peculiar structure of the brain disposes to madness; but what that peculiar structure is, has never been demonstrated: it may also be readily presumed, that a peculiar structure of the brain may be generated, as well as of feature and limb." Again, the same author remarks, "Of all the hereditary diseases, madness is supposed to be the most constant and persevering; for, even if one generation escape, the taint is presumed to cling to the succeeding branches, till, either by admixture with a purer stock, or by education or management, it is neutralized or drained away."

Case of a Remarkable Failure of Memory.—The following extraordinary case was published in an American work many years ago. The patient was a clergyman, about thirty years of age; a man of learning and acquirements, who, at the termination of a severe illness, was found to have lost the recollection of everything, even the names of the most common objects. His health being restored, he began to acquire knowledge just as a child does. After learning the names of objects, he was taught to read, and after this, began to learn the Latin language. He had made considerable progress when, one day in reading his lesson with his brother, who was his teacher, he suddenly stopped, and put his hand to his head. Being asked why he did so, he replied, "I feel a peculiar sensation in my head; and now it appears to me that I knew all this before." From that time he rapidly recovered his faculties. A state of the mental faculties somewhat analogous occasionally occurs in diseases of simple exhaustion. Many years ago (says Dr. Abercrombie) I attended a lady who, from a severe and neglected diarrhœa, was reduced to a state of great weakness, with remarkable failure of her memory. She had lost the recollection of a particular period, of about ten or twelve years. She had formerly lived in another city, and the period of which she had lost the recollection was that during which she had lived in Edinburgh. Her ideas were consistent with each other, but they referred to things as they stood before her removal. She recovered her health after a considerable time, but remained in a state of imbecility resembling the dotage of old age.

Lunacy.—It often happens that persons may converse for some time with a lunatic, and find him apparently composed and rational; he will discuss the floating topics of the day as another man; accord with the most enlightened on the general principles of morals, and correctly estimate the light and shadow of human conduct. If the observer should here retire, he might be convinced of his sanity; but let him protract the discourse—let him touch the fatal string which throws his mind into discord—let him draw the hair-trigger which inflames the combustible materials of his disease, and he will be surprised, if not alarmed, at the explosion. The sweeping tyranny of madness scorns the demarcation which limits the sober mind; and it should likewise be taken into account, that the subjects which constitute the insanity of a person, are the prominent features of his mind, and are more frequently recurred to than any other. It is true we may discuss ordinary topics like other men; but this to him is a species of by-play, and he soon reverts to the interest or catastrophe of his drama. Whatever may be the subject of discourse, and however rationally he may appear to treat it, the experienced practitioner will expect, and he will not often be disappointed, to find that by some unaccountable association, even ordinary topics are linked with his darling delusion,—the mass of his mind will point out that the smallest rivulet flows into the great stream of his derangement. —*Haslam.*

